# Medical Report, Laid before the Annual General Meeting of the Governors of the Hospital for Small-Pox; Held June 3, 1824

**Published:** 1824-08

**Authors:** George Gregory

**Affiliations:** 8, Upper John-Street, Golden-Square


### STATISTICAL MEDICINE.

ART. I.—*Medical Report, laid before the Annual General Meeting of the Governors of the Hospital for Small-Pox; held June 3, 1824.*

MY LORDS AND GENTLEMEN,

THE exertions of your medical officers for the cure and prevention of small-pox, have been rewarded, during the last year, by a degree of success, which, compared with the results of former years, it is most gratifying to your Physician to report.

The total number of in-patients admitted during the year 1823, amounted to 151; of whom, 114 were discharged cured, and only 37 died,—being very nearly the lowest rate of mortality which the records of your Hospital present for the last forty years. In the first five months of the present year, 72 patients have been admitted; of whom 19 have died,—being in the somewhat increased ratio of 25 in 100. The remainder have been either discharged cured, or may now be seen in the wards of the Hospital, convalescent. One-third of those who have died since the commencement of the present year, exhibited in their bodies all those extreme marks of violence and malignity of disease, which, precluding from the very first all hopes of relief, have rendered the small-pox, in every age of the world, an object of such disgust and dread.

The arrangements which have been made with the guardians of the poor in several of the parishes of the metropolis, (particularly St. Giles in the Fields,) have caused an unusual number of infants and children to be admitted lately within your walls; and your Physician is sorry to state, that a very large proportion of the mortality has fallen upon that helpless class of patients. Fourteen children under seven years of age (one-fifth of the total admissions,) have been received into the house since the 1st of January of the present year; and of them, eight have died.

Reverting to the proceedings of the year 1823, your Physician has to report, that, of the 151 patients treated within its walls during that period, forty-seven had previously been vaccinated. Of these, in the ordinary course of the disease, and without the protecting influence of vaccination, twelve, at the very lowest computation, would have died. It must, then, be highly satisfactory to the Governors to learn that the *whole of these persons* have been restored to society. It is not, indeed, to be denied that seven of them suffered severely under the attack of the disorder, and that it required from four to six weeks for *their* perfect cure; but it is no light praise of vaccination to say, that, even when imperfectly performed, (and there was reason to presume that such had been the case in most of these instances,) it still had the power of mitigating the horrors of this dreadful disease, and of preserving life, though it proved insufficient to resist the inroads of the contagion.

Of the 72 persons admitted since the commencement of this year, two had been inoculated for the small-pox, and bore the marks of having already once passed through the disease. Nineteen had been vaccinated; and it is the painful duty of your Physician to report, that of them one died, without mitigation of the symptoms in any stage of the disorder. The case was that of a young woman, vaccinated, when an infant, in the country; and your Physician took pains to ascertain the exact mode in which the process had been conducted. This he was happily enabled to do, with the assistance of a professional gentleman residing on the spot, whose inquiries tended to establish, most satisfactorily, that vaccination had in this instance been performed imperfectly and unskilfully, and that no reasonable confidence could at any time have been placed in it. The melancholy result of the case, however, will not be without its use, if it impresses upon all those who are engaged in vaccinating, the indispensible necessity of a close attention to every stage of that process, upon which the safety of the individual in after-life so immediately depends.

Your Physician turns with pleasure to notice, that, in the year 1823, 3129 persons were vaccinated, under his superintendence; and that, in the first five months of the present year, 1280 have passed through the same protecting process. With most unfeigned satisfaction he can add, that at no period, since the discovery of vaccination, has the confidence of the lower orders of people in this town, in the security which it is capable of affording, appeared to him greater than at present. It ought well to be considered, too, that no persons have such good opportunities of judging; for the contagion of small-pox, which visits provincial towns only at intervals, is always present in London, seeking whom it may devour. Those, indeed, whom a few unfavourable cases have impressed with an undue sense of the imperfections of the vaccine influence, would receive an useful lesson by attending at your Hospital, at the hours appropriated to vaccination. They would then learn to appreciate, in the grateful acknowledgments of thousands, the true value of that great blessing, which it was the glory of JENNER to have diffused;—and, though occasional failures will undoubtedly occur, to warn us, like spots upon the sun, that nothing is perfect, yet, to the eye that contemplates, on a wide scale, the results of vaccination, these are lost in the brilliancy of its general career. No where has it been more severely tried,—but no where has it come out of the trial more triumphant, than in the annals of your useful and excellent Charity.

GEORGE GREGORY, M.D.

8, *Upper John-street, Golden-square;*
*June 3, 1824.*

